# How Saprolegniales became successful parasites

**DOI:** 10.1371/journal.ppat.1014359

**Published:** 2026-07-02

**Authors:** Paula Ortega-López, Javier Diéguez-Uribeondo

**Affiliations:** Department of Mycology, Real Jardín Botánico (RJB-CSIC), Madrid, Spain; Tufts Univ School of Medicine, UNITED STATES OF AMERICA

## What are the Saprolegniales?

The Saprolegniales (Oomycota) are eukaryotic filamentous microorganisms that belong to the SAR supergroup (Stramenopiles, Alveolata, and Rhizaria), which includes numerous economically and ecologically relevant organisms [1]. Traditional estimates suggested that the order comprises between 150 and 250 species [2]. However, our recent survey of Mycobank and the literature since 1970 identifies approximately 300 described species within the order. This number likely represents an overestimate, as many taxa have not been rediscovered since their original description. Moreover, molecular sequence data is currently available for only about 120 species (approximately 40%), leaving a significant portion of their diversity in taxonomic uncertainty.

## Are all of Saprolegniales species parasites?

**Fig 1:**
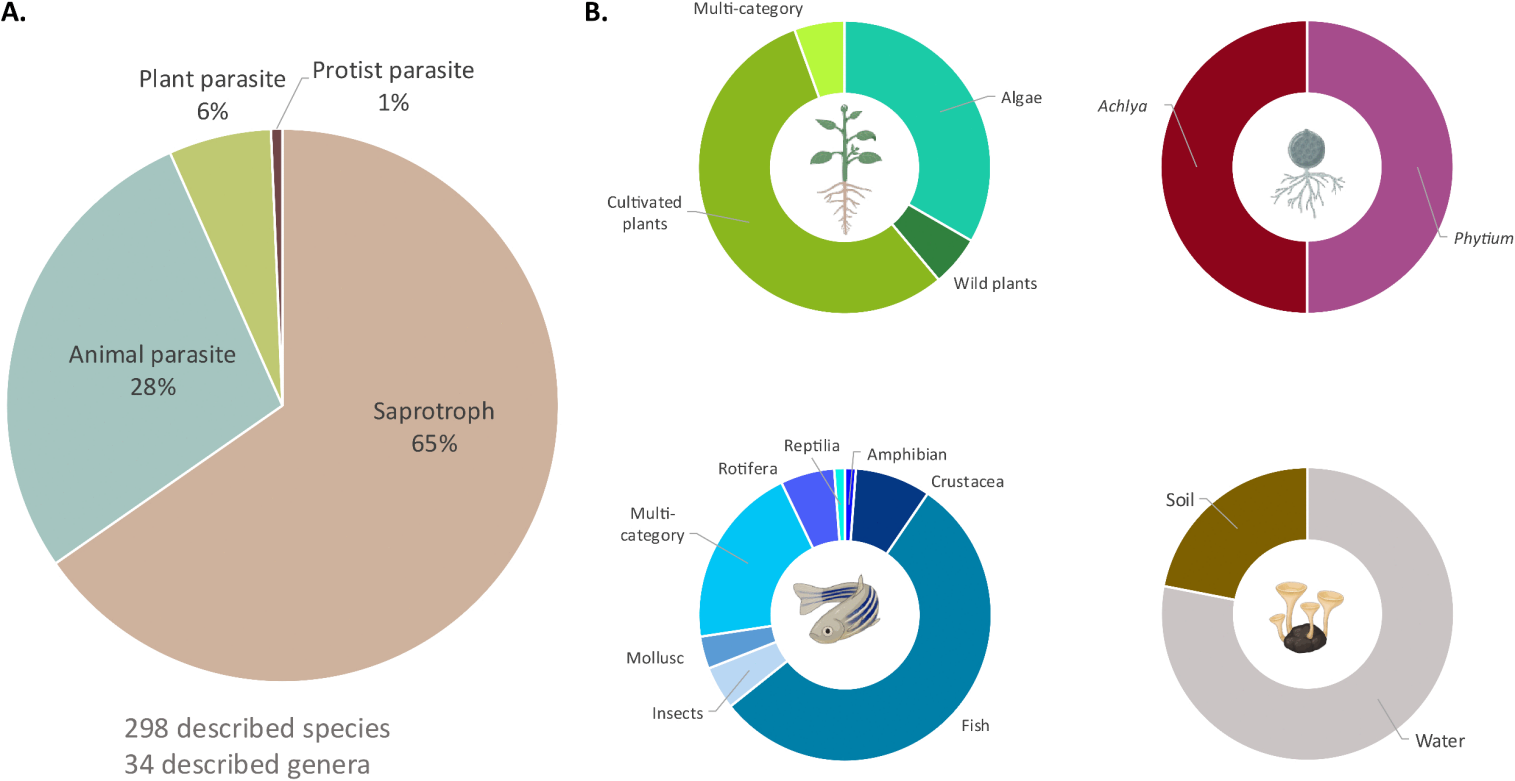
Diversity of lifestyles within the order Saprolegniales. Data synthesized from Mycobank (2026) and an extensive literature review of freshwater parasites. **(A)** Distribution of lifestyles across 298 validly described species. **(B)** Proportional distribution of ecological niches within the order: Plant parasites (upper left), Protist parasites (upper right), Animal parasites (lower left), and Saprotrophs (lower right). Multi-category means there is evidence for infection in two groups or more.

Despite these taxonomic gaps, the ecological roles of Saprolegniales are diverse and impactful. Traditionally regarded as key saprotrophs in aquatic and wet terrestrial ecosystems [3] our review reveals that 65% of the species are classified as saprotrophic (see Fig 1), though this figure may be overrepresented as saprotrophy is often the “default” classification in the absence of proven parasitism. This group plays key ecological roles [2] including parasitic species responsible for emerging infectious diseases in both plants and animals. Many of them are spreading at a global scale causing substantial economic losses in wildlife, agriculture, and aquaculture worldwide [4].

Parasitic Saprolegiales infect a broad diversity of hosts, including plants, algae, invertebrates, vertebrates as well as other protists (see Fig 1). Specifically, *Saprolegnia parasitica* and *Aphanomyces astaci* are among the most devastating animal pathogens within the group and rank among the best-studied oomycete due to their role in severe and emerging diseases [1].

Host specialization ranges go from highly specific parasites to broad-spectrum opportunists. The genus *Aphanomyces* exemplifies high host specificity, with species like *A. astaci* and *A. cochlioides* showing restricted host ranges for both animal and plant hosts, respectively [5]. Conversely, *S. parasitica* and related species, while restricted to freshwater fish hosts, display broader within-group host range [6], infecting a broad range of freshwater fish species while retaining saprotrophic capabilities that enable survival on decaying organic matter when live hosts are unavailable. On the same way, many saprotrophic species act as opportunistic parasites in suitable conditions like wounded hosts or poor water quality [7]. This suggest Saprolegniales have a high plasticity to change their lifestyle strategy.

## How did parasitism evolve in Saprolegniales?

Although saprotrophic lifestyles are common within the Saprolegniales, the evolutionary emergence of parasitism in this order still remains an important unresolved question. Parasitic lifestyles occur across all Oomycota lineages with several parasitic species representing deep phylogenetic branches [8]. Early-diverging groups primarily parasitize marine algae and invertebrates showing most likely a marine environment origin [9]. This observation also supports the hypothesis proposed by Beakes and colleagues (2012), which posits that adaptation to parasitism is deeply embedded in the lineage, whereas saprotrophy is derived trait. Genomic analyses of the secretomes of parasitic (e.g., *Achlya hypogyna*) and non-parasitic (e.g., *Thraustotheca clavata*) oomycetes further support this view [10]. Importantly, the evolution of these lifestyles has been strongly influenced by horizontal gene transfer (HGT). Evidence suggests that oomycetes acquired key metabolic and effector genes from fungi and bacteria, facilitating the colonization of diverse hosts and environments [11]. Furthermore, this evolutionary proximity to the fungal kingdom is evidenced by a convergent evolution in intercellular communication; both groups have evolved analogous pathways for pheromone precursor exchange to trigger sexual development [12]. Despite these insights, the evolutionary origin of parasitism within the Saprolegniales remains unresolved, particularly concerning the transition between diverse host niches and when these transitions occurred during the diversification of the lineage.

## How do Saprolegniales infect their hosts?

**Fig 2:**
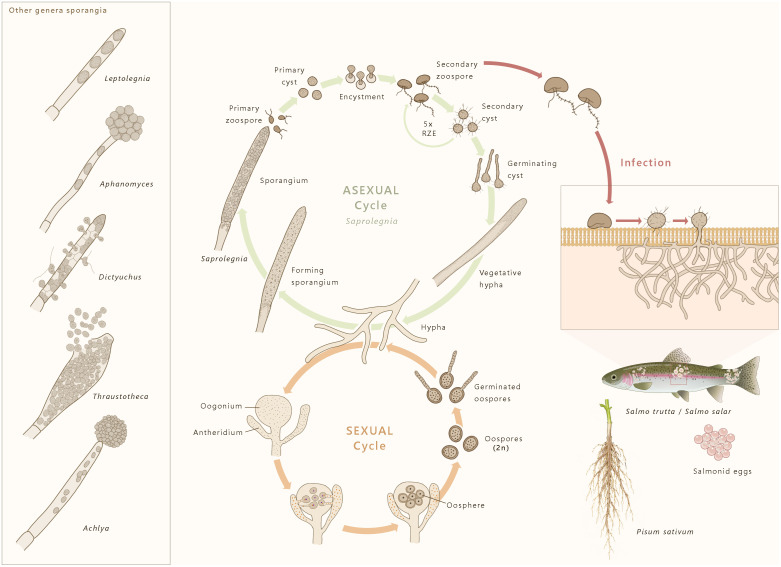
Lifecycle in Saprolegniales. Schematic representation of both sexual an asexual reproduction, illustrating the developmental transitions and key morphological phases of the organism. (Left) Taxonomic diversity of the sporangia of different genera of Saprolegniales represented. (Right) Infection process with potential hosts depicting the transition from encystment to host penetration. Illustration by Marina Trillo Gabaldón.

The colonization of hosts and the success of parasitic strategies in Saprolegniales rely on virulence factors that are expressed and at different stages of their life cycle [13]. Despite differences in host specificity, plant and animal pathogenic oomycetes share common infectious features, such as the production of specialized structures and spores (sporangia, zoospores, cysts, and oospores) and convergent infection strategies [7]. For this reason, understanding their life cycle, which includes both sexual and asexual reproduction, is essential (see Fig 2). Asexual reproduction allows the dissemination and transmission. The main route of transmission is through secondary zoospores which, due to their high motility and their ability to respond to electrochemical and chemotactic signals, locate convenient points of infection on the host [14]. Once a suitable host or substrate is reached, the secondary zoospores attach and rapidly encyst [4]. Initial host attachment is facilitated by specialized morphological adaptations; for instance, secondary cysts of *S. parasitica* possess long hooked hairs that significantly increase attachment efficiency to fish tissues [15]. After attaching to the host via a secondary cyst, zoospores can either initiate infection or re-emerge as motile zoospores if attachment fails. This process, known as polyplanetism or repeated zoospore emergence (RZE), appears to maximize infection opportunities. These cycles of encystment and re-emergence has been observed up to six times [14], but the zoospores can persist in the cyst state short periods of time. These transitions ultimately determine how far zoospores can travel and remain infective.

Sexual reproduction, in contrast, contributes to genetic exchange and survival under unfavorable or extreme conditions [12]. It produces thick-walled oospores capable of persisting in hostile environments, often referred to as “resistance oospore”, which ensure long-term survival of the species. These structures represent the best-known resistance form in oomycetes and are comparable to those extensively studied in *Phytophthora*, where oospores can persist in soil for up to four years [16].

Beyond life-cycle strategies, additional virulence factors contribute to pathogenic success. For instance, *S. parasitica* possesses one of the largest repertoires of proteases described in any organism, including serine proteases capable of degrading fish IgM, thereby neutralizing the host’s humoral immune response [13]. In plant-infecting species, some parasites, such as *Aphanomyces euteiches,* secrete modular proteases and cell-wall-degrading enzymes into the plant apoplast during infection, targeting structural components of tissues rather than immune proteins [17]. Beyond physical tissue degradation, Saprolegniales actively manipulate host immunity through diverse secretome components. Recent genomic and biochemical evidence highlights their ability to suppress or bypass innate immune responses via specialized enzymes [18]. These enzymes interfere with host chemical signaling during infection through unique structural adaptations in their active sites. Similarly, crayfish proteinase inhibitors effectively block extracellular proteinases from *Aphanomyces spp.*, illustrating reciprocal molecular arms-race dynamics [19]. Together, these molecular tools, alongside RxLR and CRN effectors, in both, plant and animal parasites, and an extensive repertoire of proteases capable of degrading host immunoglobulins and host tissues [13,20], reflect the specialized evolutionary strategies that Saprolegniales have developed to establish successful infections across a broad range of hosts.

## How do Saprolegniales disperse across large geographic distances?

Another important factor influencing pathogenic success is dispersal. Although short-distance dispersal is well understood, uncertainty remains regarding how these species colonize new water bodies, particularly when they are separated by geographical barrier and large distances between terrestrial habitats, such as oceanic islands. Beyond the classical view of water-borne zoospore movement, the role of biotic vectors has gained attention as a potential driver of Saprolegniales dispersal. A well-documented example is the spread of the crayfish plague in Europe, where invasive crayfish species (e.g., *Procambarus clarkii, Pacifastacus leniusculus* or *Faxonius limosus*) act as chronic carriers and active vectors of *A. astaci*, facilitating its expansion across disconnected watersheds [21]. This biological transport suggests that the movement of Saprolegniales is not limited to water currents but may also be closely linked to the mobility of their hosts, associated fauna or resistant spores, i.e., oospores. Based on this principle, it has been hypothesized that dipterans or other winged insects could act as dispersal vectors. Their mobility, saprophagous habits, and frequent interactions with moist substrates make them plausible candidates for transporting oospores or secondary cysts. Similarly, the potential role of waterfowl in the mechanical transmission of oomycete parasites has received increasing attention, as these birds can connect geographically isolated water bodies [22].

## What are the key unanswered questions?

Despite the extensive research on the pathogenicity of specific model species [1], several critical gaps remain about how Saprolegniales evolved into successful parasites. Regardless of evidence suggesting an ancestral marine origin for parasitism in oomycetes, the role of HGT and fungi gen convergence complicates this narrative. It remains unclear whether their path to animal parasitism as it seems in Saprolegniales, and the divergence toward plant parasitism in Peronosporales, represents a linear progression or a series of independent, derived events enabled by genomic plasticity. Addressing this question could reveal whether parasitic abilities are inherited from an ancestral parasitic oomycete (implying a widespread latent capacity for parasitism). Another unresolved issue is how the traits that enable successful infection evolved. Although several virulence factors and infection strategies have been described in model pathogens, the evolutionary origins and diversification of structures such as the attachment structures, *e.g.,* cysts hooked hairs of *S. parasitica*, as well as the molecular mechanisms underlying pathogenicity, remain poorly understood across the order. Finally, the processes enabling the global spread of these parasites remain uncertain. Although zoospore motility explains local transmission, the mechanisms allowing Saprolegniales to cross large geographical barriers remain largely unknown. In particular, the role of biotic vectors (such as aquatic animals, dipterans, or waterfowl) or resistant spores, *i.e.,* oospores, in long-distance dispersal deserves further attention, especially in the context of environmental change and emerging diseases. Addressing these questions would provide insights into both the evolutionary origins of pathogenicity, the identification of virulence factors, disease control and management of emerging diseases cause by parasitic Saprolegniales.
